# Electromyographic activity of the masseter muscle in individuals with group function and canine guidance

**DOI:** 10.34172/joddd.2021.038

**Published:** 2021-12-05

**Authors:** Nathália de Oliveira Domingos, Roberto Bernardino Júnior, Patrícia Teixeira de Carvalho Gaspar, Frederico Balbino Lizardo, César Ferreira Amorim, Daniela Cristina de Oliveira Silva

**Affiliations:** ^1^Faculty of Dentistry, Federal University of Uberlândia, Uberlândia, Brazil; ^2^Department of Human Anatomy, Institute of Biomedical Sciences, Federal University of Uberlândia, Uberlândia, Brazil; ^3^Master’s and Doctoral Programs in Physical Therapy, University City of São, São Paulo, Brazil

**Keywords:** Dental occlusion, Electromyography, Mandible, Masseter muscle, Mastication

## Abstract

**Background.** There is no general consensus in restorative dentistry about which lateral guidance should be established. Some studies have shown that canine guidance decreases the tension of masticatory muscles. Others have reported that group function might achieve a better physiologic distribution of occlusal forces. Also, some reports have shown that both guidances are equally acceptable. Despite all discussions, clinical evidence of one guidance being superior to another is limited. Thus, this study aimed to analyze the electromyographic (EMG) activity of masseter muscles in individuals with group function and canine guidance.

**Methods.** Twenty volunteers of both genders, aged 20-25, were divided into two groups: GF (group function guidance, n=10) and CA (canine guidance, n=10). EMG activity of masseters was captured using surface electrodes during habitual maximum intercuspation (HMI) and right and left lateral jaw movements and recorded using EMG amplitude values (RMS – root means square). Student’s t-test was used to compare mean RMS values between the groups and lateral movements in each group.

**Results.** During HMI, there was no difference in masseter EMG activity between the groups. Both masseters showed higher activity in group GF only on the right side during lateral movements, while the left masseter exhibited higher activity on the nonworking side in both groups. The activity of both masseters distributed by tooth was higher in group CA.

**Conclusion.** During tooth restorative procedures, any guidance is acceptable considering HMI. However, group function guidance is more favorable during lateral movements due to greater dissipation of occlusal pressures.

## Introduction

The tooth action during mastication occurs not only with simple vertical movements of opening and closing but also with various excursive lateral and anterior movements. These movements determine each function, food crushing, and breaking, enabling and making it easier for the digestive system to digest and absorb foods.^1^ In individuals with normal occlusion, mastication is characterized by dental contacts, a pause in maximum intercuspation position, and broad lateral movements.^2^ Lateral movements can be performed by two guidance types, i.e., canine and group function.^1^ During lateral jaw movements in canine guidance, the lower canine cusp incisal border slides on the upper canine palatal surface until the two canines touch.^3^ However, during group function guidance, this movement occurs along with molar and premolar teeth.^4^ The side toward which the canine tooth slides is called the “working side,” and the other side is named the “nonworking side”.^1,3,4^

Canine lateral guidance is a standard of disocclusion guidance, in which only canines should be in touch.^3,4^ In the cervical-incisal direction, as canines are the longest teeth in the dental arch, the other teeth must be in disocclusion. Consequently, the working-side upper and lower canines make a sliding dental contact, enabling nonworking-side disocclusion.^3,4^ This occlusal scheme was first described by D’Amico,^5^ who stated that canines guide the mandible during eccentric movements when antagonistic teeth are in functional contact, and the proprioception of the canine periodontal receptors is more sensitive, causing a decrease in the tension of masticatory muscles.

Group function guidance is a disocclusion guidance pattern in which there is a partial touch of canine and premolar teeth or total touch of canine, premolar, and molar teeth during lateral jaw movements.^3,4^ This lateral movement will promote a disocclusion of other nonworking-side teeth.^3,4^ According to Beyron,^6^ working-side group function establishes a physiological distribution of occlusal pressures, leading to a small probability of causing bruxism.

Considering occlusion principles, when rehabilitating a patient, the establishment of canine guidance is recommended, in which only canines must touch each other, and all other teeth must be in disocclusion.^1^ In the absence of canine guidance, group function is chosen, involving working-side premolars and molars.^3^ Any other dental touch can cause occlusal instability, with consequences both on the teeth directly involved and on others at a distance, in addition to temporomandibular joint (TMJ) injury.^1,3^ Some authors defend the establishment of group function guidance since that canine guidance does not offer comfort and no better distribution of beneficial loads for the periodontium, nor does it provide a better masticatory efficiency.^7,8^ On the other hand, Miralles^9^ reported that canine and group function guidances are both equally acceptable when restoring dentition. This evidence supports a flexible principle of occlusion rather than a preconceived occlusion theory.

Although each lateral disocclusion theory has its supporters, clinical evidence of one model being superior to the other is limited.^10^ Instead of following a preconceived philosophy of lateral occlusion types, it is worth questioning the influence of lateral occlusion on the patient’s comfort and their physiological masticatory system.^10^

To compare each theory, the masseter muscle activity has been used as a reference since it is an important muscle involved in mastication and lateral jaw movements.^11-15^ Muscle performance in mastication can be assessed by objective tests of masticatory efficacy and/or performance and subjective tests of masticatory ability.^16-20^

Different methods to evaluate muscle performance in mastication can have different results. Among the various methodologies, surface electromyography, which is an instrument for clinical and kinesiological evaluation of muscle function, is an advanced technology to quantify total muscle work activity, in addition to estimating muscle fatigue and power simultaneously.^21-23^

Electromyographic (EMG) activity of masticatory muscles was studied in patients with patterns of canine and group function guidances, with divergent results.^12,24,25^ While Mizutani et al^25^ found that patients with canine guidance had nearly half of the muscle activity recorded in patients with group function guidance, other authors did not observe any significant differences in the activity of the masticatory muscles between the groups of canine and group function guidances, suggesting that both occlusion patterns can be used to treat patients that have lost their lateral guidance.^24,26^

In this context, this study aimed to analyze the EMG activity of the masseter muscle in individuals with group function and canine guidance to verify if muscle activity differs between the two guidance types. This question can resolve any doubts about the recommendation of reestablishing the canine or group function guidance and in which situations each guidance works better, whether in the habitual maximum intercuspation (HMI) or the lateral sliding (lateral movement).

## Methods

### Sample

Twenty students, male and female, 20 to 25 years of age, were recruited from the Dentistry School of the Federal University of Uberlandia, Uberlandia, Minas Gerais, Brazil. After the anamnesis and clinical examination, the volunteers were distributed to two groups: GF (group function guidance, n = 10) and CA (canine guidance, n = 10).

The inclusion criterion for group GF was bilateral group function guidance with canine/first premolar/second premolar disocclusion, with bilateral canine guidance with canine disocclusion alone for group CA. Also, all the volunteers had neutro-occlusion (Angle class 1). Exclusion criteria for both groups were based on a history of any joint or neuromuscular injuries that could influence muscle activity; volunteers with mixed lateral guidance, total or partial removable prosthesis, and those with missing canine, premolar, or molar teeth; individuals with overjet and overbite outside scientifically accepted standards or with any occlusal interference.

### EMG instruments

EMG activity was captured using two disposable surface electrodes (Medpax MP-43 adult; DBI Comercio e Importação LTDA, São Paulo, SP, Brazil) that were coupled to a differential bipolar pre-amplifier receiver (EMG System do Brasil LTDA, São José dos Campos, SP, Brazil) with 20-times gain, 10 GΩ input impedance, and common mode of the rejection ratio >120 db.

EMG signs were recorded on a computerized electromyograph with analogical-digital converter, 16-bit resolution with ± 2 V range, 2000-times amplifier total gain, Butterworth filters of 20-500 Hz bandpass, and sample rate frequency of 2 kHz per channel (EMG 830 C, EMG System do Brasil LTDA), and further processed using the EMGLab V1.1 version 2014 software of the same manufacturer.

### EMG procedures

Data collection was performed in a single step for both groups. The volunteers were placed on a backboard chair with a straight body. The skin over both masseter muscles was shaved and cleaned with 70% alcohol. Then, the electrodes were positioned precisely in the midline of the muscle belly between the motor point and the muscle tendons,^27^ with an interelectrode distance of 20 mm, according to the manufacturer’s recommendations. A ground electrode was positioned on the frontal bone to improve the conductivity, minimizing any interference. The placement of the electrodes was performed considering each individual’s biotype specificity and according to the European guidelines for surface electromyography (SENIAM).^28^

**Figure 1:**
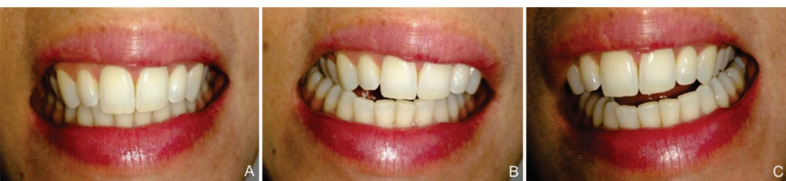
Situations in which the electromyography signals were captured. A: Habitual maximum intercuspation. B: Final position of the right lateral movement (representative photo of an individual with group function guidance). C: Final position of the left lateral movement (representative photo of an individual with canine guidance).

**Figure 2:**
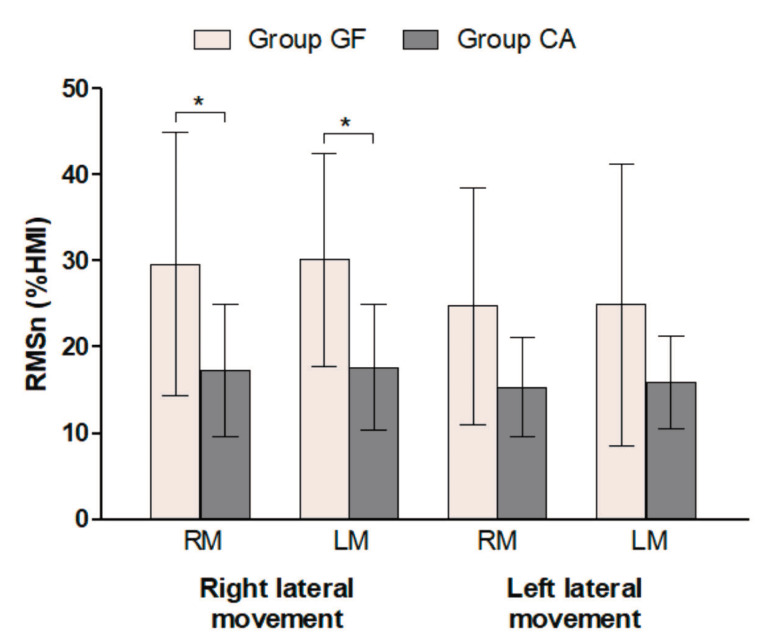
RMS (root mean square) normalized values (RMSn) obtained from the right masseter (RM) and left masseter (LM) muscles during right and left lateral movements in individuals with group function (Group GF) and canine (Group CA) guidances. The bars represent the mean and standard deviation obtained from 10 volunteers. * *P* < 0.05, as determined by the Student t test.

**Figure 3:**
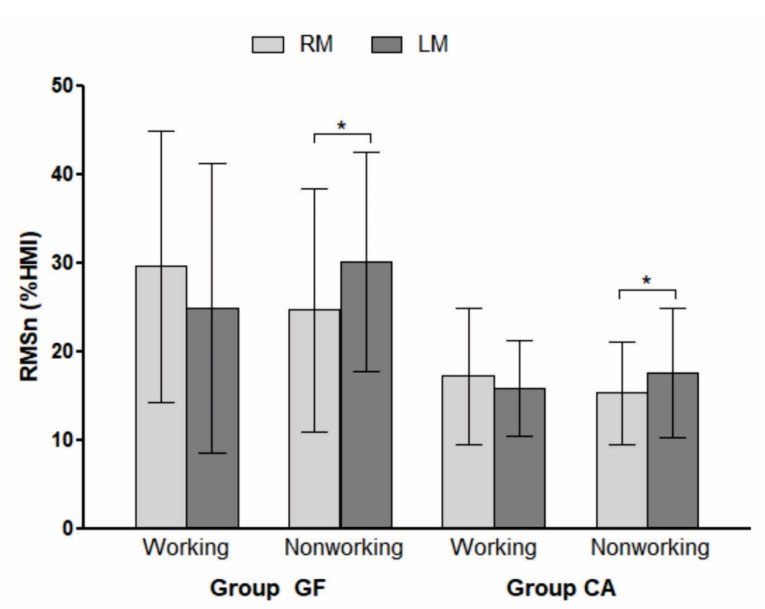
RMS (root mean square) normalized values (RMSn) obtained from the right masseter (RM) and left masseter (LM) muscles during lateral movements toward the working- and nonworking-sides in individuals with group function (Group GF) and canine (Group CA) guidances. The bars represent the mean and standard deviation obtained from 10 volunteers. * *P* < 0.05, as determined by the student *t* test.

The EMG signal was captured in three conditions: (1) HMI (Figure 1A); (2) sliding to the right side from the centric positioning - right lateral movement (Figure 1B); (3) sliding to the left side from the centric positioning - left lateral movement (Figure 1C). These conditions were recorded during a 4-sec time, with lateral movement performed for 2 sec from the centric to the canine position. The same time was used for returning to the centric position. All the contractions were monitored by a metronome (set to 1 b.s-^1^). Three sequences of each condition were performed, with a 20-second interval between sequences and a 40-second interval between conditions. During lateral movements, the contact between the upper and lower teeth was checked using a mirror, in which the individuals visualized the execution of the movement; there was no contact between the teeth at the nonworking side as all the volunteers had normal occlusion. It is worth mentioning that all the individuals were academics of the Dentistry School and, therefore, knowledgeable of guidances and lateral movements.

### Data analyses

EMG signs were recorded during the whole time of collection (4 sec) using electromyographic amplitude values (RMS – root mean square), expressed as µV. The mean of RMS values obtained from three sequences performed in each condition (HMI, right and left lateral movements) was calculated for analysis.

Raw RMS values obtained during HMI were used for comparison between the groups (GF and CA). Raw RMS values were normalized (RMSn) as a percentage of raw RMS values obtained during HMI during lateral movements (%HMI).

A comparative analysis was performed during lateral movements considering the number of teeth involved in each guidance, i.e., canine guidance (1 tooth) and group function guidance (3 to 5 teeth). Thus, mean RMSn values obtained for each muscle in the GF group were divided by the number of teeth involved, and then the mean of 10 individuals was calculated.

### Statistical analysis

Statistical analysis was performed using GraphPad Prism (GraphPad Software version 5.0, Inc. San Diego, CA, USA). UnpairedStudent’s *t*test was used to compare the means of (1) raw RMS values obtained during HMI between groups; (2) RMSn values obtained during lateral movements between groups, (3) between the right and left lateral movements in each group, and (4) considering the number of teeth involved in each guidance. All the results were considered statistically significant at a 95% significance level (*P* < 0.05).

## Results

Table 1 shows the descriptive statistics of the raw RMS values obtained from the right and left masseter muscles during HMI in individuals with group function and canine guidance. Considering the right masseter muscle, mean RMS values showed no significant difference between groups GF and CA (*P* = 0.2610). The same was observed for the left masseter muscle (*P* = 0.5037).

**Table 1:** Descriptive statistics of raw RMS (root mean square) values (µV) obtained from the right (RM) and left masseter (LM) muscles during habitual maximum intercuspation (HMI) in individuals with group function (Group GF; n = 10) and canine (Group CA, n = 10) guidances

	**Mean±Standard Deviation**	**Minimum**–**Maximum**
Group GF		
RM	132.90 ± 68.18	69.27–233.80
LM	109.50 ± 42.74	66.59–177.80
Group CA		
RM	103.30 ± 42.78	47.58–189.50
LM	97.70 ± 33.99	47.60–155.10

The mean RMSn values obtained during right and left lateral movements in groups GF and CA are presented in Figure 2. During right lateral movement, the right masseter in group GF showed significantly higher electrical activity than in group CA (29.64 ± 15.25% HMI and 17.27 ± 7.64% HMI, respectively, *P* = 0.0341); RMSn values of the left masseter during the same movement were also significantly higher than those in group CA (30.13 ± 12.33% HMI for group GF and 17.67 ± 7.27% HMI for group CA, *P* = 0.0310). No significant difference was found between the two masseters during the left lateral movement when groups GF and CA were compared.

EMG activities of the masseter muscle during lateral movements toward the working and nonworking sides in each group (GF and CA) are presented in Figure 3. On the working side, no significant difference was found in the mean RMSn values when the right and left masseter muscles were compared in group GF (29.64 and 24.93% HMI, respectively) and group CA (17.27 and 15.88% HMI, respectively). However, on the nonworking side, the left masseter presented significantly higher electrical activity than that obtained for the right masseter in both groups: GF (30.13 and 24.76% HMI, respectively, *P* = 0.0233) and CA (17.67 and 15.37% HMI, respectively, *P* = 0.0393).

In group GF, during right lateral movement, the mean RMSn values distributed per tooth were 7.52 ± 3.10% HMI for the right masseter and 7.71 ± 1.96% HMI for the left masseter muscle. During left lateral movement, these values were 6.19 ± 2.51% HMI and 6.32 ± 3.37% HMI for the right and left masseter muscles, respectively.

**Figure 4:**
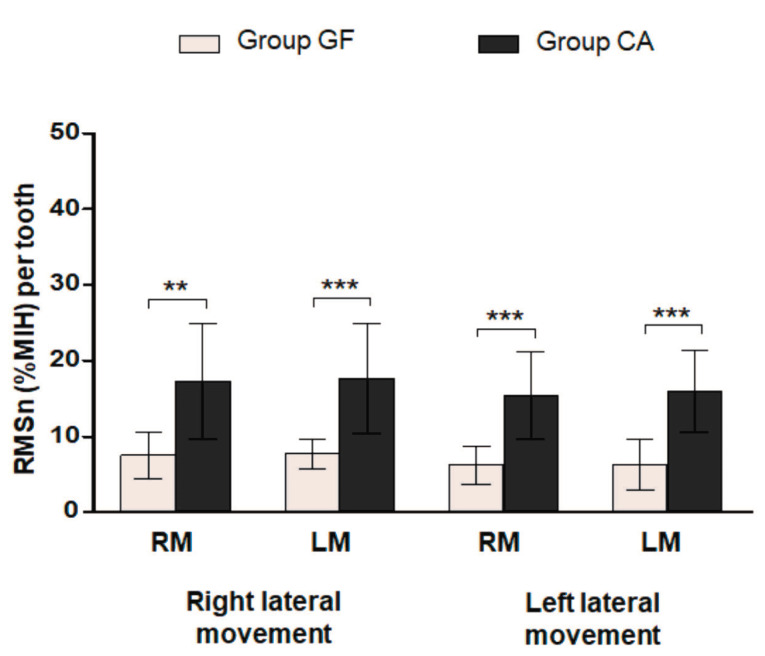
RMS (root mean square) normalized values (RMSn) obtained from right masseter (RM) and left masseter (LM) muscles divided by the number of teeth involved during left and right lateral movements in individuals with group function (Group GF) and canine (Group CA) guidances. The bars represent the mean and standard deviation obtained from 10 volunteers. ** *P* < 0.005 and *** *P* < 0.001, as determined by the student *t* test.

Figure 4 shows the comparison between the mean RMSn values obtained from masseter muscles divided by the number of teeth involved during right and left lateral movements in individuals with group function and canine guidance. The electrical activity of both muscles distributed by tooth during both movements was significantly higher for group CA (*P* = 0.001 for the right masseter during right lateral movement; *P* = 0.0006 for left masseter during right lateral movement; *P* = 0.0002 for both masseters during left lateral movement).

## Discussion

This study evaluated and compared the EMG activity of masseter muscles during HMI and lateral jaw movements in individuals with group function and canine guidance, showing significant differences between them. During HMI, although the electrical activity of the masseter muscles was lower in individuals with canine guidance (20% for the right masseter and 10% for the left one) than those with group function guidance, this difference was not statistically significant. Thus, in the statistical approach, both groups can be considered similar, suggesting that guidance does not define a pattern for rehabilitation in the HMI. However, when analyzing the mean activity of masticatory muscles over an occlusal device during clenching, Shupe et al^29^ concluded that individuals with canine guidance had a significantly lower activity than that of individuals with group function guidance. A similar result was reported by Shinogaya et al,^30^ who found a significantly lower activity of the masseter and the anterior and posterior temporal muscles during clenching in individuals with canine guidance than those with group function.

A comparison of the RMSn values obtained during lateral jaw movements showed a higher EMG activity of the masseter muscle in individuals with group function guidance, suggesting that this muscle keeps the mandible in a closed mouth state and, therefore, it is more active the closer the mandible gets to the maxilla.^22,31,32^ On the other hand, canine disocclusion produced a lower RMSn value since, in this situation, the masseter muscle is more stretched, leaving the mandible further away from the maxilla due to the canine cusp, which is the tooth with the largest clinical crown (cervical-incisal direction). This makes the masseter muscle more relaxed, producing a low electrical activity than the group function guidance, where the cusps are small and bring the mandible closer to the maxilla.

Belser and Hannam^33^ observed the same results in their study, in which individuals with canine guidance showed reduced activity of mandible elevator muscles during parafunctional clenching. Manns et al^11^ also observed a decrease in the activity of the masseter and anterior temporal muscles during lateral excursive movements in individuals with canine guidance. Furthermore, Okano et al^13^ reported that the canine guidance was associated with lower EMG activity of masticatory muscles during clenching at lateral occlusion positioning. However, Akören and Karaağaçlioğlu^12^ observed no significant difference between any guidance types, although individuals with canine guidance showed reduced activity of the anterior temporal muscle during lateral movement compared to those with group function guidance. All these convergent data on the lower EMG activity of the masseter in individuals with canine guidance can be explained by the extension of the canine clinical crown. Thus, the canine guidance presents a laterality model that can decrease the masseter muscle tension compared to the group function guidance.

In this context, all the authors above support rehabilitation by canine guidance. Other authors^1,5,34,35^ also defend the use of canine guidance due to several factors, including a favorable load on the posterior teeth, large and voluminous roots, bone reinforcement, palatal concavity, the position of the dental arch, steep cusps, greater sensitivity of the teeth, periodontal receptors for proprioception, and decreased masticatory muscle tension.

In contrast, Borromeo et al^36^ found no differences between RMS values of the masseter muscles in individuals with canine and group function guidances during lateral jaw movements using interocclusal devices. These discrepant results are possibly due to the non-similar methodologies such as habitual occlusion and device guides.

Although individuals with canine guidance present less electrical activity in the masseter muscle, it is worth discussing that this value is distributed over one tooth. In contrast, the values are distributed over two or more teeth in individuals with group function guidance. In addition, Alexander^37^ reported that the canine tooth is not necessarily the strongest one since the molars (together) have at least four roots and provide great support for the other teeth; the author also reported that the canine tooth cannot support all the occlusal pressures as a preventive measure to protect the other teeth, as they are subjected to the same aspects of periodontal disease injury as other teeth. In this sense, it is noteworthy that it is more reasonable for the group function to protect the canine tooth than the opposite.

During the right lateral movement, the left masseter in individuals with group function guidance had to increase its activity to protect the TMJ, corresponding to the load applied by the right masseter. On the other hand, in individuals with canine guidance, the left masseter exhibited no significant contraction, as it is naturally the most active muscle, and there was not much work to carry out TMJ-protecting contraction. During left lateral movement, the left masseter in individuals with group function guidance had a normal contraction. In contrast, in individuals with canine guidance, the contraction increased in 20% of the cases, as they have a naturally more active left masseter muscle. Such observations on the protective contraction of joints are consistent with the findings of Aquino et al.^38^

An analysis of the masseter EMG activities during the lateral movements toward the working and nonworking sides in each group showed a higher electrical activity by the left rather than the right masseter muscle only on the nonworking side, which can be clarified by the same protective contraction of the TMJ explained above. Since most individuals in both groups had mastication preference on the right side, the right masseter was naturally more active. Then, on the nonworking side, the left masseter required greater activity than the right masseter to protect TMJ. Therefore, the left masseter required more work to perform protective contraction of TMJ during right lateral movements, while the right masseter required less work to perform TMJ-protecting contraction during left lateral movements. Accordingly, the stability of various joints is not exclusively dependent on the passive structures of these joints but also on a neuromuscular mechanism that regulates the protective action of the muscles.^38^

Considering the EMG activity of the masseter muscles distributed over the tooth during lateral movements, individuals with canine guidance exhibited electrical activity approximately 40‒45% higher than individuals with group function guidance. These data suggest that group disocclusion seems favorable for the dissipation of occlusal pressures applied by the masseter muscle. This question is due to the number of teeth involved in group disocclusion during lateral movements, ranging from 2 to 5 teeth. Thus, to compensate for the use of the canine guidance, which involves only one tooth, the RMSn value for these individuals should be 2 to 5 times lower than that of an individual with group function guidance. Therefore, considering the teeth involved, the group function guidance is capable of causing greater dissipation of the occlusal pressure applied by the masseter muscles. In addition, premolars and molars involved in the group function guidance have a larger occlusal surface than the canine cusp. Therefore, they are larger teeth to support and spread occlusal pressures.

This analysis corroborates a study by Schuyler,^7^ who reported that the group function guidance is the most responsible for the correct dissipation of occlusal pressures. Alexander^39^ showed that canine teeth could not support excessive functional loads, causing vertical bone loss, and the use of group function guidance was preferable for better dissipation of occlusal pressures. Beyron^6^ reported that the group function on the working side establishes a physiological distribution of occlusal pressures, which is less likely to cause bruxism. Jemt et al^8^ reported that the group function disocclusion pattern has a greater degree of movement and greater mandibular velocity than canine disocclusion; therefore, it is better to use the group function guidance. Alexander,^37^ Butler and Zander,^24^ and Yaffe and Ehrlich^40^ also defend the use of the group function guidance, as it is natural like the canine guidance, and that the canine tooth should work with the other teeth, and not as an independent entity.

On the other hand, some authors^9,41,42^ reported that canine guidance or group function guidance are equally acceptable, and it is difficult to establish a straight pattern for occlusion rehabilitation. They stated that instead of adhering to a preconceived occlusion scheme when restorative treatment is indicated, a simple, conservative, and practical occlusion scheme that allows for aesthetic treatment should be considered.^43^ Additionally, before the treatment, one must consider the patient’s type of occlusion, periodontal status, TMJs, musculature, and the cranium-cervical component.^26^ Another author^44^ raises doubts about which type of disocclusion is the best during oral rehabilitation, as there is no scientific support to establish specific guidance that can evaluate the risk or benefit of having different patterns of occlusal contact during lateral excursions. Considering the studies above, the findings of the present research are divergent since group disocclusion was shown to be preferable to the dissipation of occlusal pressures due to the higher number of teeth involved in lateral movements, the large occlusal surfaces, and the greater number of roots (molar) that assist in supporting the loads applied by the masticatory muscles.

Finally, it is noteworthy that surface electromyography can capture the activities of deep muscles, together with the superficial ones, depending on their thickness or even adjacent muscles. Thus, a limitation of this study might be that the activity of other facial muscles, like the pterygoids, has been recorded together with the masseter activity, even though it is the most superficial muscle. Another limitation was the lack of criteria for the formation of groups considering the different types of the facial skeleton (dolichocephalus, brachycephalus, and mesocephalus). Thus, it is suggested in future research to include the analyses of different types of the facial skeleton and the two phases of the lateral movement separately, as well as evaluations of other masticatory muscles and intra- and inter-sex comparisons.

## Conclusion

In conclusion, any lateral guidance (canine or group function) is acceptable during tooth restoration considering HMI. The masseter muscle on the nonworking side increases its activity to protect TMJ in both lateral guides during lateral movements. Additionally, canine guidance can reduce masseter muscle tension; however, considering the number of teeth involved in lateral jaw movements, group function guidance is more favorable to restoring the dentition, as it achieves greater dissipation of occlusal pressures.

## Authors’ contributions

NOD and PTCG carried out data curation, investigation, and formal analyses. RBJ and FBL contributed to conceptualization; visualization, writing, methodology, and supervision; CFA and DCOS carried out project administration, writing, reviewing, and editing. All the authors read the final manuscript and revised it.

## Acknowledgments

We would like to thank the National Council for Scientific and Technological Development (Conselho Nacional de Desenvolvimento Científico e Tecnológico – CNPq, Brazil) for support this research

## Funding

Not applicable.

## Competing interests

The authors declare no competing interests with regards to the authorship and/or publication of this article.

## Ethics approval

This study was approved by the Human Research Ethics Committee of the Federal University of Uberlandia (project CAAE: 57307716.3.0000.5152, opinion 1.733.683). Each volunteer signed an informed consent form.
